# Geographic Distribution and Future Projections of Mild Cognitive Impairment and Dementia in Greece: Analysis from 1991 to 2050

**DOI:** 10.3390/brainsci15060661

**Published:** 2025-06-19

**Authors:** Themis P. Exarchos, Konstantina Skolariki, Vasiliki Mahairaki, Constantine G. Lyketsos, Panagiotis Vlamos, Nikolaos Scarmeas, Efthimios Dardiotis, on behalf of the Hellenic Initiative Against Alzheimer’s Disease (HIAAD)

**Affiliations:** 1 Bioinformatics and Human Electrophysiology Laboratory, Department of Informatics, Ionian University, 49132 Corfu, Greece; exarchos@ionio.gr (T.P.E.); vlamos@ionio.gr (P.V.); 2 Institute of Digital Biomedicine, Ionian University Research and Innovation Center, 49132 Corfu, Greece; 3 Department of Psychiatry and Behavioral Sciences, Johns Hopkins University School of Medicine, Baltimore, MD 21205, USA; kskolar1@jh.edu (K.S.); kostas@jhmi.edu (C.G.L.); 4 Department of Genetic Medicine, Johns Hopkins University School of Medicine, Baltimore, MD 21205, USA; vmachai1@jhmi.edu; 5 Johns Hopkins Bayview Medical Center, Baltimore, MD 21224, USA; 6 First Department of Neurology, Aiginition Hospital, School of Medicine, National and Kapodistrian University of Athens, 11528 Athens, Greece; ns257@cumc.columbia.edu; 7 Taub Institute for Research in Alzheimer’s Disease and the Aging Brain, The Gertrude H. Sergievsky Center, Columbia University, New York, NY 10032, USA; 8 Department of Neurology, Medical School, University of Thessaly, 41334 Larisa, Greece

**Keywords:** dementia, mild cognitive impairment, distribution heatmaps, future projection

## Abstract

Background: Greece is among the fastest-aging countries globally, with one of the highest proportions of elderly individuals. As a result, the prevalence of mild cognitive impairment (MCI) and dementia is among the highest in Europe. The distribution of affected individuals varies considerably across different regions of the country. Method: We estimated the number of people living with MCI or dementia in Greece and visualized these estimates using heatmaps by regions for four census years: 1991, 2001, 2011, and 2023 (the 2023 census was delayed due to the COVID-19 pandemic). Age- and sex-specific prevalence rates of MCI and dementia were obtained from the Hellenic Longitudinal Investigation of Aging and Diet. These prevalence rates were then applied to population data from each census to estimate the number of affected individuals per region. Results: There was a consistent increase in the number of people living with MCI, rising from 177,898 in 1991 to 311,189 in 2023. Dementia cases increased from 103,535 in 1991 to 206,939 in 2023. Projections based on future census data for 2035 and 2050 suggest that the number of people with MCI will reach 375,000 and 440,000, respectively, while dementia cases will increase to 250,000 in 2035 and 310,000 in 2050. Conclusion: Given that each person with dementia typically requires care from at least two caregivers over time, these projections highlight the profound impact the dementia epidemic will have on Greece. The heatmaps developed in this study can serve as valuable tools for policymakers in designing and implementing clinical care programs tailored to the needs of each region based on the projected burden of disease.

## 1. Introduction

Greece is experiencing a rapid demographic shift driven by a rapidly aging population. The population fraction aged ≥65 years is among the highest globally, positioning Greece as one of the fastest-aging countries in Europe [1]. This demographic trend has significant implications for public health, particularly concerning age-related cognitive disorders such as MCI and dementia. Understanding the regional distribution of individuals in Greece living with these conditions is crucial for effective healthcare planning and resource allocation. MCI is often considered an intermediary stage between normal cognitive aging and dementia, marked by noticeable cognitive decline that does not significantly interfere with daily activities. Individuals with MCI have significant condition-specific healthcare needs and are at an increased risk of progressing to dementia, therefore presenting an opportunity for prevention. Dementia is a syndrome characterized by a decline in memory, thinking, behavior, and the ability to perform everyday activities. Dementia, with Alzheimer’s disease being the most common form, is a leading cause of disability and dependency among older adults worldwide. Globally, approximately 55 million people were living with dementia in 2020, with this number projected to reach 139 million by 2050 [2,3].

In Greece, the prevalence of MCI among individuals aged 65 and older is estimated at 13.11%, while the prevalence of dementia is approximately 5% in the same age group [4]. These figures underscore the substantial burden of cognitive impairment within the aging Greek population. However, there are limited data examining the regional distribution and temporal trends of MCI and dementia across the country’s regions. Such information is vital for identifying areas with higher burdens and for tailoring public health interventions accordingly.

In the Greek population, the major causes of MCI and dementia align with both global and local demographic and health trends. Advancing age is the most significant risk factor, with increasing age sharply raising the likelihood of both conditions. Low educational attainment also plays a critical role, with fewer years of schooling associated with higher cognitive impairment. Vascular and lifestyle-related factors—such as hypertension, diabetes, obesity, physical inactivity, and poor dietary habits—are prevalent in Greece and contribute notably to cognitive decline. On the genetic front, the *APOE* ε4 allele is the most well-established risk factor, significantly increasing the odds of both MCI and dementia. Greek studies, particularly from the HELIAD cohort, have shown that individuals carrying this allele are at greater risk of progression from MCI to dementia. Recent research has also identified a polygenic risk score associated with white matter disease as a strong genetic predictor of MCI and Alzheimer’s disease, especially in older adults with low education. Although large-scale investigations of familial genetic mutations (e.g., *APP*, *PSEN1*, *PSEN2*) [5] have not yet been conducted in Greece, current findings highlight a multifactorial risk profile—where age, education, lifestyle, and genetics interact to shape dementia outcomes.

This study aims to estimate the total number of individuals living with MCI or dementia in Greece during four census years—1991, 2001, 2011, and 2023—and to visualize their regional distribution through heatmaps. Rather than focusing on prevalence rates, the analysis highlights the absolute patient count in each area. We also provide projections for 2035 and 2050 based on demographic trends. As a comprehensive assessment of how the number of affected individuals has changed over time and across regions, this study is intended to support policymakers and healthcare providers in designing targeted strategies to manage the increasing burden of cognitive impairment in Greece’s aging population.

## 2. Materials and Methods

This study utilized data from two primary sources:

1. Hellenic Longitudinal Investigation of Aging and Diet (HELIAD): A population-based study that provided prevalence rates for MCI and dementia, stratified by sex and age categories (65–69, 70–74, 75–79, 80–84, 85+) [4];
2. Hellenic Statistical Authority (ELSTAT): Census data from 1991, 2001, 2011, and 2023 (delayed from 2012 due to COVID-19 delays), detailing the population distribution by age, sex, and region [1].

We developed a cross-sectional estimate for each census year to estimate the number of individuals with MCI and dementia in each region. Additionally, we estimated longitudinal projections for the years 2035 and 2050, based on ELSTAT demographic forecasts [6].

Prevalence rates from HELIAD were applied to the corresponding demographic groups in each census year to estimate the number of individuals with MCI and dementia (Table 1). Specifically, the prevalence rates for each age and sex category were multiplied by the respective population counts from the census data. For future projections, we applied the same prevalence rates to the projected population figures for 2035 and 2050.

Heatmaps were generated to visualize the geographic distribution of MCI and dementia across Greek regions for each census year and for the projected years. Prevalence estimates for ages 55 to 64 were obtained from [7].

## 3. Results

Table 2 displays population estimates by age and sex over time, from 1991 to 2023.

**Figure 1:**
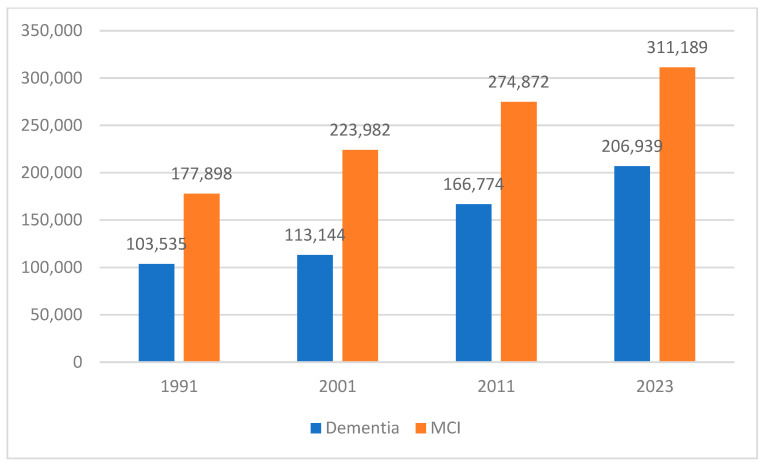
Estimation of patients with MCI and dementia over time.

Figure 1 displays national estimates of people living with MCI or dementia over time, based on the previous population estimate and HELIAD prevalence estimate.

Figure 1 presents national estimates of individuals living with MCI or dementia over time. It is important to note that actual longitudinal prevalence data across the Greek population are not currently available. This limitation stems primarily from the inherent challenges in consistently monitoring patients with cognitive impairment. Greece lacks a unified, nationwide dementia registry, and data collection remains fragmented across hospitals, regional health authorities, primary care services, and day care centers. Moreover, many cases of MCI and early-stage dementia remain undiagnosed or unreported, especially in rural or under-resourced regions. As a result, prevalence estimates must rely on cross-sectional population studies, such as HELIAD, combined with census data to model trends over time.

**Figure 2:**
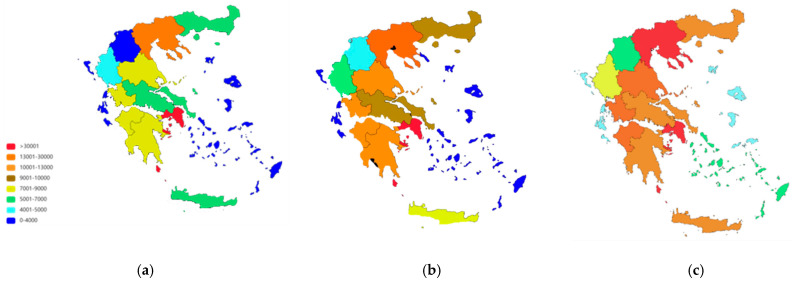

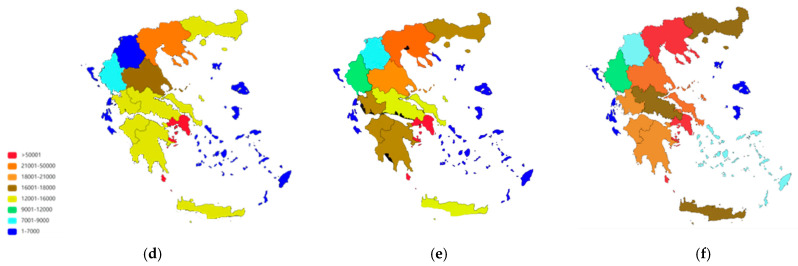
Distribution of people living with MCI or dementia over time in the different regions of Greece: (**a**) dementia in 2001, (**b**) dementia in 2011, (**c**) dementia in 2023, (**d**) MCI in 2001, (**e**) MCI in 2011, (**f**) MCI in 2023.

Figure 2 displays the distribution of people living with dementia or MCI by region and its evolution over time, for 2001, 2011, and 2023 (for 1991, only summary data were available from the ELSTAT census).

Table 3 presents estimates of populations for Greece in 2035 and 2050, from [6].

**Figure 3:**
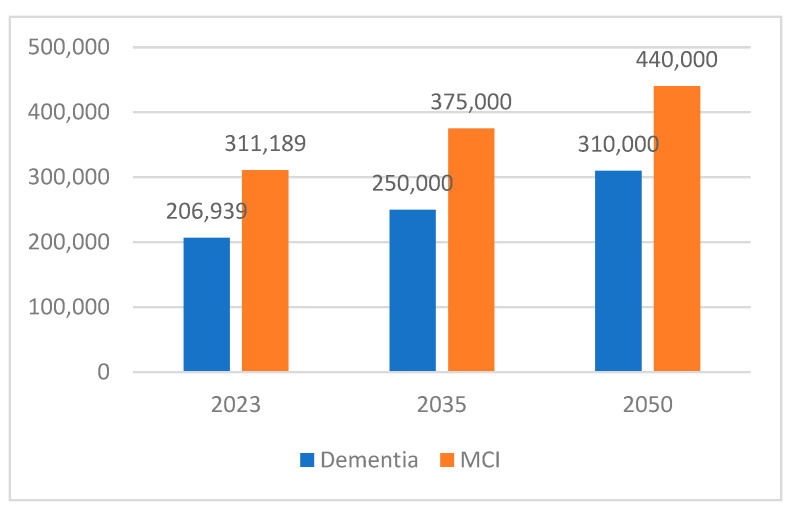
Projections for people living with dementia or MCI in 2035 and 2050 along with the estimation for 2023.

Based on the above table, with the same prevalence numbers from HELIAD, projections for 2035 and 2050, along with the previous estimates, are displayed in Figure 3.

**Figure 4:**
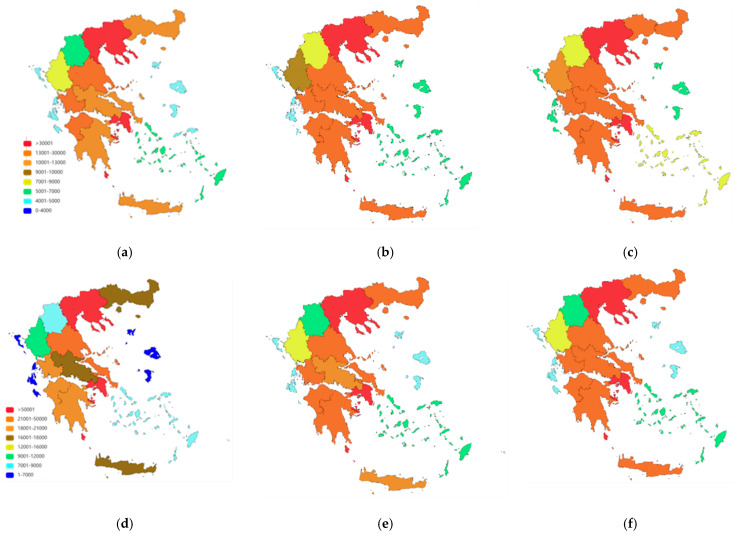
Distribution of population counts with MCI or dementia overtime in the different Greek regions: (**a**) dementia in 2023, (**b**) dementia in 2031, (**c**) dementia in 2050, (**d**) MCI in 2023, (**e**) MCI in 2035, (**f**) MCI in 2050.

Finally, based on the population distribution in the different Greek regions and the above projection, heatmaps for 2035 and 2050 are shown in Figure 4.

## 4. Discussion

This study estimates the regional distribution and temporal evolution of MCI or dementia in Greece, examining trends from 1991 to 2023 and projecting future scenarios to 2035 and 2050. These analyses indicate a substantial increase in both conditions over the examined period, reflecting demographic trends and highlighting regions with particularly significant burdens. The findings underscore the urgency for targeted healthcare planning and policy formulation to manage the expected growth of people living with MCI and dementia anticipated to exert considerable pressure on Greece’s health and social care systems.

The observed trend of higher prevalence of MCI and dementia in certain male age groups, as reported in this study, reflects an interplay between biological, lifestyle, and healthcare-related factors [8]. While overall dementia prevalence tends to be higher in females—partly due to their longer life expectancy—specific age brackets may show elevated rates among males, particularly in mid-to-late adulthood (e.g., ages 65–74). This pattern has been noted in other population-based studies and may relate to men’s higher burden of vascular risk factors, such as hypertension, diabetes, smoking, and cardiovascular disease, which are closely linked to vascular cognitive impairment and mixed dementia. Additionally, men in these age groups may be less likely to seek early medical advice or engage in health-promoting behaviors, potentially leading to underdiagnosis at earlier stages and higher prevalence at later ones. In contrast, women often live longer and may accumulate risk for Alzheimer’s-type dementia later in life. The sex differences observed in MCI prevalence may also suggest gender-related differences in cognitive reserve, social engagement, and exposure to occupational or educational experiences that influence cognitive aging. These findings highlight the importance of tailoring prevention strategies and screening programs not only by age but also by sex, accounting for the distinct risk profiles and care needs of men and women.

The near doubling in MCI cases—from approximately 178,000 in 1991 to over 311,000 in 2023—and in dementia cases—from around 104,000 in 1991 to nearly 207,000 in 2023—align closely with Greece’s rapidly aging population profile [1,4]. The regional variations observed reveal significant geographical disparities. Regions with historically higher proportions of older residents demonstrate a steeper increase in numbers. This spatial heterogeneity emphasizes the necessity for region-specific healthcare strategies rather than nationwide uniform interventions.

The projections for 2035 and 2050 indicate an alarming upward trajectory. By 2050, the number of individuals affected by dementia and MCI will approach 310,000 and 440,000, respectively. Such projections, though contingent upon stable prevalence rates and demographic trends, highlight a future health crisis if preventive and mitigating measures are not promptly implemented. Given the anticipated increase in the number of older citizens due to improving life expectancy, addressing this health challenge requires immediate and proactive health-policy interventions at local and national levels.

The projected increases in the number of people living with MCI and dementia pose critical challenges for Greece’s healthcare infrastructure and caregiving capacity. Each dementia or MCI patient typically requires significant long-term supportive care from multiple caregivers—often family members—placing substantial economic, social, and emotional burdens on households [9,10,11]. Considering that the current care infrastructure in Greece primarily relies on family-based informal caregiving, this increase in counts will undoubtedly stress caregiving capacities thereby undermining societal productivity and well-being.

An important strength of this study lies in its use of sex- and age-stratified prevalence data across multiple census years, allowing for an in-depth examination of temporal trends in MCI and dementia [12]. By disaggregating the data by sex, it becomes possible to identify whether the observed increases over time are uniform across male and female populations or if distinct patterns emerge. For instance, if the prevalence of MCI has increased more rapidly among males in certain age brackets, this might reflect higher exposure to modifiable vascular risk factors or lower engagement with preventive healthcare services. Conversely, a steeper increase in dementia prevalence among older women may be attributable to their longer life expectancy and increased survival into high-risk age categories. Evaluating these sex-specific trends over time can also help validate whether changes reflect true increases in disease burden versus improved diagnosis or shifts in population structure. Such granular analysis enhances the predictive power of the study and supports the development of targeted interventions, tailored not only by region and age but also by sex. Future research should continue to monitor these trajectories to ensure that public health responses remain both evidence-based and responsive to emerging disparities.

Relevant healthcare services—such as specialized memory clinics, daycare centers, and long-term care facilities—remain unevenly distributed across Greece. By estimating the absolute number of individuals affected by MCI and dementia in each region, the heatmaps offer practical guidance to policymakers in identifying areas with the highest patient loads [13]. Prioritizing these high-burden “hotspot” regions for targeted resource allocation—such as establishing new memory clinics and implementing caregiver training programs—could substantially improve care and support for individuals and their families. Additionally, public health initiatives focused on awareness, prevention (e.g., healthy diet, physical activity, cognitive stimulation), and early detection may help reduce the future burden of cognitive decline.

The trends identified in Greece closely mirror European and global patterns. Globally, dementia prevalence will nearly triple by 2050, driven largely by population aging [14]. Similar projections have been reported in other Mediterranean and Southern European countries, characterized by longevity and demographic aging, including Italy and Spain [3]. However, regional variations within Greece remain distinctive and reflect unique local demographic shifts, including internal migration, urbanization, and economic disparities. This suggests that national policies must incorporate flexible approaches, addressing both demographic factors and socioeconomic determinants of health that vary across regions.

This study presents certain limitations. Our projections rely on stable age-specific prevalence rates of dementia and MCI derived from HELIAD. Potential changes in risk factor profiles, healthcare interventions, or diagnostic practices may alter actual prevalence [15]. For example, improvements in cardiovascular health or early diagnosis or new treatments may affect either incidence or prevalence rates, affecting long-term projections. Competing risks and age-related changes in projected survival may affect dementia projections. Census data are subject to limitations, including underreporting and variations in data collection methodologies, especially notable during the COVID-19 pandemic period. Similarly, epidemiological studies are also subject to limitations when attempting to accurately estimate dementia prevalence. Our reliance on a single prevalence dataset (HELIAD) could introduce additional biases towards either overdiagnosis or underestimate prevalence if unreported or undiagnosed cases differ from studied cohorts.

In addition, while the findings of this study underscore the urgency of preparing for a potential dementia pandemic in Greece, it is equally important to consider the rapidly evolving landscape of dementia research and treatment. Predictive models based on current prevalence rates and demographic projections are invaluable for policy planning, yet they inherently assume static conditions in disease incidence and care availability [16]. In reality, major scientific advances—particularly in the development of disease-modifying therapies and early detection—may significantly alter these trajectories. Immunotherapies, such as anti-Aβ monoclonal antibodies, have recently shown promise in slowing disease progression in early-stage Alzheimer’s disease, though questions remain regarding their long-term efficacy, accessibility, and cost [17]. Similarly, the emergence of plasma biomarkers like phosphorylated tau (e.g., *p-tau217*) is revolutionizing early diagnosis, enabling detection before clinical symptoms appear [18]. As these innovations become integrated into clinical practice, they may reduce the burden of advanced dementia, shift the age of onset, or improve outcomes through earlier intervention. Therefore, while the mathematical projections in this study provide critical insight into future needs, they should be interpreted as dynamic estimates—ones that must be revisited regularly in light of therapeutic and diagnostic progress. A flexible, forward-looking policy approach will be key to bridging current projections with future realities.

Future studies should explore longitudinal cohort designs to capture incidence rates and identify modifiable risk factors specific to Greek populations. Detailed investigation into socio-economic, dietary, genetic, and lifestyle factors may enhance understanding of regional variations observed in dementia prevalence [19]. Moreover, developing and regularly updating a national dementia registry will improve surveillance accuracy and assist policymakers in real-time health planning.

From a policy perspective, immediate actions should prioritize resource allocation towards under-resourced high-risk regions, caregiver support initiatives, and nationwide awareness campaigns [20]. Integrating dementia-friendly practices into urban planning and community services will also become increasingly crucial. Ultimately, this study provides a clear roadmap for healthcare stakeholders, policymakers, and researchers, highlighting the necessity of strategic and region-sensitive responses to Greece’s growing dementia challenge.

## 5. Conclusions

This study highlights the accelerating burden of MCI and dementia in Greece, driven by rapid population aging and regional demographic disparities. Projections indicate a significant rise in affected individuals by 2050, underscoring the urgency for proactive, region-specific healthcare planning. The uneven distribution of care infrastructure and reliance on informal caregiving risk overwhelming families and the health system. Targeted resource allocation, public health initiatives, and the development of specialized services are essential to mitigate future impacts. Establishing a national dementia registry and investing in preventive strategies will be critical for sustainable management. These findings provide a data-driven foundation for informed policy action at both local and national levels.

## Figures and Tables

**Table 1:** Prevalence of MCI and dementia in Greece, by age and sex, from HELIAD.

Age	Prevalence of MCI			Prevalence of Dementia		
Years	♂	♀	Total	♂	♀	Total
65–69	12.00%	8.40%	10.10%	3.00%	0.80%	1.90%
70–74	10.20%	10.20%	10.20%	3.50%	1.80%	2.40%
75–79	16.70%	16.70%	16.70%	6.80%	6.80%	6.90%
80–84	12.10%	18.30%	15.70%	11.20%	16.30%	15.40%
85+	25.70%	7.90%	14.50%	11.40%	31.60%	21.40%
≥65	13.90%	12.40%	13.00%	6.10%	8.90%	7.60%

**Table 2:** Population estimates by age and sex over time.

	1991			2001			2011			2023		
	M	F	T	M	F	T	M	F	T	M	F	T
55–59	322,468	332,641	655,109	271,095	289,120	560,215	321,466	338,902	660,368	461,932	310,043	771,975
60–64	308,403	336,362	644,765	298,181	341,893	640,074	301,589	324,180	625,769	441,046	303,671	744,717
65–69	210,101	243,705	453,806	291,600	331,645	623,245	241,832	266,444	508,276	395,478	270,848	666,326
70–74	150,599	193,396	343,995	247,136	297,882	545,018	246,264	295,901	542,165	349,537	248,378	597,915
75+	257,197	349,245	606,442	278,765	380,205	658,970	441,729	616,682	1,058,411	722,215	580,110	1,302,325

**Table 3:** Population projection for Greece, in terms of 65+ and 85+ for 2035 and 2050.

		Total Population	65+	85+
	Census 1951	7629.7	522.4	30.8
	1/2015 (ELSTAT)	10,858.0	2269.0	303.2
	1/1/2020	10,718.6	2386.2	377.0
Baseline projections	1/1/2035	10,104.6	2853.0	483.5
	1/1/2050	9503.1	3208.8	667.2

## Data Availability

Data are derived from public domain resources. The data presented in this study are available in the repository of the Hellenic Statistical Authority at https://www.statistics.gr/en/home/.
